# Solid State Polyselenides and Polytellurides: A Large Variety of Se–Se and Te–Te Interactions

**DOI:** 10.3390/molecules14093115

**Published:** 2009-08-24

**Authors:** Christian Graf, Abdeljalil Assoud, Oottil Mayasree, Holger Kleinke

**Affiliations:** Department of Chemistry, University of Waterloo, Waterloo, ON, N2L 3G1, Canada

**Keywords:** selenium, tellurium, bonding, hypervalent

## Abstract

A large variety of different interactions between the chalcogen atoms, *Q*, occur in the solid state structures of polyselenides and polytellurides, including both molecular and infinite units. The simplest motifs are classical *Q*_2_^2–^ dumbbells and nonlinear *Q_n_*^2–^ chains (*n* = 3, 4, 5, ..), e.g. found in alkali metal polychalcogenides. In addition, nonclassical so-called hypervalent motifs exist in the form of linear *Q*_3_^4–^ units or within larger units such as *Q*_4_^4–^ and *Q*_5_^4–^. Infinitely extended *Q* units include zigzag, *cis*/*trans* and linear chains, as well as planar and slightly puckered layers. Several of those are susceptible to Peierls distortions, leading to the formation of both commensurate and incommensurate superstructures and anomalies in transport properties, including metal-nonmetal transitions.

## 1. Introduction

Solid state materials based on chalcogenides, *i.e.*, sulfides, selenides and tellurides, play a large role in today’s society. Examples include semiconductor devices, e.g. in solid state electronics [1], fast-ion conductors [2,3], rechargeable batteries [4], data storage including phase-change materials [5,6,7], chalcogenide glasses [8], and the thermoelectric energy conversion [9,10,11,12]. Polychalcogenides, like the potential thermoelectric material HfTe_5_, are materials that comprise homonuclear bonds between negatively charged chalcogen atoms, e.g. a Se–Se bond within Se_2_^2–^ pairs of Rb_2_Se_2_ [13]. These bonds occur in chalcogen-rich materials, where the chalcogen atom cannot be reduced to attain the closed-shell formation as in *Q*^2–^. For example, Se carries a charge of –1 in Rb_2_Se_2_, like O in SrO_2_, and therefore forms one Se–Se bond. As such, Rb_2_Se_2_ is a typical Zintl phase. Zintl phases *AX*_x_ consist of an electropositive element *A* (here: Rb) and a more electronegative element *X* of the later (post transition) main groups (here: Se). Assuming a complete charge transfer of all of *A*’s valence-electrons to *X*, the cation *A^z^*^+^ possesses a full octet, and the (formal) anion *X^z/x^*^-^ attains the octet by forming homonuclear *X*–*X* bonds in addition to the reduction by *A*. In most cases, these bonds are classical single bonds (two-center two-electron, *2c-2e*), i.e. exactly one bond is formed for each electron missing to complete the octet of *X* [14,15,16].

The rich structural chemistry in particular of the polyselenides and polytellurides is the scope of this review, going beyond the common *2c-2e* bonds: hypervalent interactions as found in XeF_2_ [17] or SF_6_ [18] are often observed in various fragments of these materials [19]. In addition, weaker fractional bonds or cohesive interactions of different lengths, as in elemental tellurium, render the Se/Te substructures intriguingly complex [20,21], which in turn may be beneficial for the thermoelectric energy conversion [22]. Four reviews about polychalcogenides from the years 1995 to 2000 underline the importance of this field [20,23,24,25].

## 2. Results and Discussion

### 2.1. Molecular units in polyselenides and polytellurides

#### 2.1.1. Oligomeric *Q_n_*^2–^ motifs

**Figure 1:**
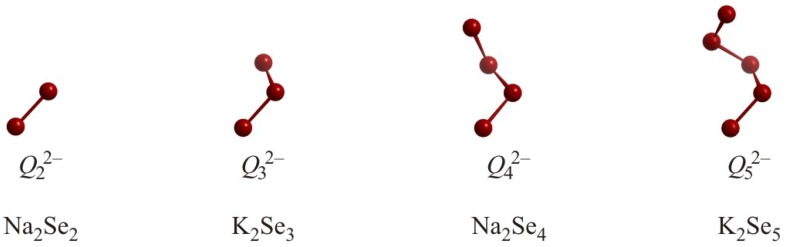
From left to right: Oligomeric *Q_n_*^2–^ motifs in Na_2_Se_2_, K_2_Se_3_, Na_2_Se_4_, and K_2_Se_5_.

The simplest polychalcogenide motif is the dumbbell unit *Q*_2_^2–^, occurring for example in Na_2_Se_2_ [26], Rb_2_Se_2_ and Rb_2_Te_2_ with Se–Se and Te–Te distances of 2.38 Å, 2.47 Å and 2.78 Å, respectively (Figure 1). These dumbbells are also found in organic compounds with similar bond lengths, e.g. in [N(CH_3_)_4_]_2_Te_2_ (*d*_Te–Te_ = 2.74 Å) [27] and [Na(CH_3_OH)_3_]_2_Te_2_ [28]. Assigning charges is straightforward in these examples, e.g. (Na^+^)_2_Se_2_^2–^, with Se_2_^2–^ being isoelectronic to Br_2_ and I_2_, with 14 valence-electrons for the dumbbells, which are therefore held together by one *2c-2e* bond. While generally these distances compare well with the sum of the single bond radii, *r*_Se_ = 1.17 Å and *r*_Te_ = 1.37 Å [29], the influence of the cations is evident, for the Se–Se bond in Rb_2_Se_2_ is approximately 0.1 Å longer than in Na_2_Se_2_.

The dumbbells can formally be extended to oligomeric chain-like units *Q_n_*^2–^ (with *n* = 3, 4, 5, 6, 12, 13) by adding neutral *Q* atoms to the chain, wherein the terminal *Q* atoms remain negatively charged. Each neutral *Q* atom participates in two *2c-2e* bonds, comparable to the elemental structures of selenium and tellurium. Se_3_^2–^ groups (with one central neutral Se atom) occur for example in, K_2_Se_3_ [30], Sr_2_SnSe_5_ [31] and Ba_2_SnSe_5_ [32], with bond lengths of approximately 2.4 Å, and Te_3_^2–^ groups in K_2_Te_3_ with *d*_Te–Te_ = 2.80 Å [33] and in Ba_7_Au_2_Te_14_ with *d*_Te–Te_ = 2.89 Å [34]. It is evident that the lengths of regular single (*2c-2e*) bonds may vary substantially, noting that these bonds are significantly elongated compared to 2× *r*_Se_ = 2.34 Å and 2× *r*_Te_ = 2.74 Å, respectively.

The *Q*_3_^2–^ anions are bent, ideally exhibiting C_2v_ symmetry, with typical *Q*–*Q*–*Q* bond angles of 105° - 110°, in accordance with the VSEPR concept, which suggests tetrahedral arrangement of the two bonds and two free electron pairs around the central *Q* atom. The packing of the three-dimensional structure may cause a severe distortion of the angle as well, e.g. down to 92° in Ba_7_Au_2_Te_14_.

**Figure 2:**
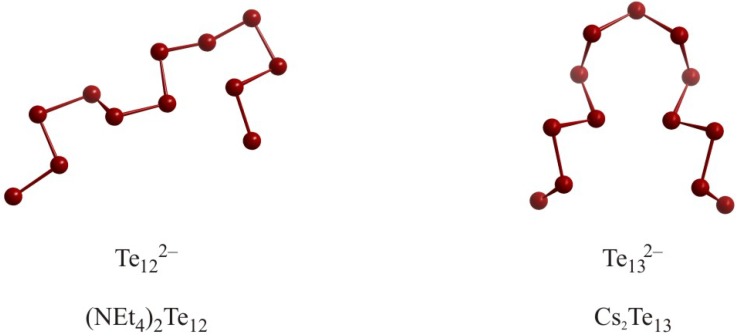
Oligomeric Te*_n_*^2–^ motifs in (NEt_4_)_2_Te_12_ (left) and Cs_2_Te_13_ (right).

Anions with larger *n*, such as Se_4_^2–^ in Na_2_Se_4_ (*d*_Se–Se_ = 2.35 Å and 2.36 Å) [35], Te_4_^2–^ in (Ph_4_P)_2_Te_4_∙2CH_3_OH (*d*_Te–Te_ = 2.72 Å and 2.76 Å) [36], Se_5_^2–^ in K_2_Se_5_ (2.34 Å ≤ *d*_Se–Se_ ≤ 2.37 Å) [37], and Se_6_^2–^ in [Me_3_N(CH_2_)_13_CH_3_]Se_6_ (2.27 Å ≤ *d*_Se–Se_ ≤ 2.35 Å) [38] are best described as oligomeric zigzag or helical chain fragments. Larger chain-like polychalcogenide anions, though extremely rare, do exist, like Te_12_^2–^ in [N(C_2_H_5_)_4_]_2_Te_12_ [39] and Te_13_^2–^ in Cs_2_Te_13_ [40] (Figure 2), and they are often - as in these two examples - interconnected via longer interchain interactions (here: 3.14 Å and 3.18 Å). These interactions are much shorter than twice the van der Waals radius and will be discussed later in this review.

#### 2.1.2. Oligomeric *Q_n_*^4–^ motifs 

**Figure 3:**
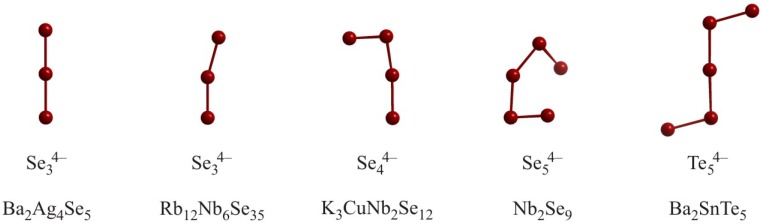
From left to right: Se_3_^4–^ (D_∞h_), Se_3_^4–^ (C_2v_), Se_4_^4–^, *cis*-Se_5_^4–^ and *trans*-Te_5_^4–^.

Since the *Q_n_*^4–^ fragments comprise two more valence-electrons than their *Q_n_*^2–^ counterparts, they cannot contain the same *2c-2e* bonds. *n* = 2 is a hypothetical case only, as it would correspond to two isolated closed-shell *Q*^2–^ anions. *n* = 3 is realized in Se_3_^4–^ units of Ba_2_Ag_4_Se_5_ [41], and Rb_12_Nb_6_Se_35_ [42]. In the former, Se_3_^4–^ exhibits the highest symmetry possible, namely linearity and equidistant interactions, thus point group D_∞h_, while Rb_12_Nb_6_Se_35_ is comprised of two differently distorted, almost linear Se_3_^4–^ units of C_2v_ and C_1_ symmetry, respectively. In these two compounds, the Se–Se–Se bond angle of Se_3_^4–^ varies between 180° and 164°, and the Se–Se bond lengths between 2.59 Å and 2.77 Å (Figure 3).

Ba_2_Ag_4_Se_5_ contains two isolated Se^2–^ anions and one Se_3_^4–^ per formula unit, according to the ionic formulation (Ba^2+^)_2_(Ag^+^)_4_Se_3_^4–^(Se^2–^)_2_. Electronic structure calculations and electrical conductivity measurements confirmed the semiconducting, hence electron precise character of this compound [41]. Rb_12_Nb_6_Se_35_ is a special case, containing both Se_3_^2–^ and Se_3_^4–^ units, according to the formula (Rb^+^)_12_(Nb^5+^)_6_(Se_3_^2–^)_2_(Se_2_^2–^)_7_(Se_3_^4–^)_3_(Se^2–^)_6_. Since diffuse reflectance measurements supported the semiconducting and thus closed-shell character of Rb_12_Nb_6_Se_35_ [42], this treatment of Rb_12_Nb_6_Se_35_ within the Zintl concept is justified.

With 22 valence-electrons, Se_3_^4–^ is isoelectronic with hypervalent linear XeF_2_ and I_3_^–^. Several examples are known that contain the linear I_3_^–^ motif, including CsI_3_ [43] and [Ph_4_As]I_3_ [44]. Ideally the two I–I distances are equivalent, with a bond angle of 180° as in [Ph_4_As]I_3_ (*d*_I–I_ = 2.90 Å), but deviations from the centrosymmetric arrangement are often found with smaller cations, like in CsI_3_ with I–I bonds of 2.84 Å and 3.04 Å and a bond angle of 178°. The linear arrangement is well understood based on Rundle’s model [45], which treats the *s* orbitals as well as the *p* orbitals of π symmetry as lone pairs. Then the frontier orbital set consists of one filled σ bonding, one filled nonbonding, and one empty σ antibonding molecular orbital, formed by the *p*_z_ orbitals when *z* corresponds to the molecular axis. The nonbonding orbital contains a nodal plane at the center of the triatomic unit, resulting in a three-center-four-electron (*3c-4e*) bond. The validity of Rundle’s model waswas - in principle - confirmed for Se_3_^4–^ via Gaussian calculations using the B3LYP functional [41].

Since the *3c-4e* bonds are electron deficient, containing only one bonding molecular orbital for two bonds (which is why they are often called "half" bonds), they are longer than the regular *2c-2e* bonds. Correspondingly, the I–I single bond in I_2_ of 2.76 Å is much shorter than the above-mentioned I–I bonds (2.84 Å and 3.04 Å), and Se–Se single bonds (2.34 Å) are shorter than the Se–Se bonds of Se_3_^4–^ (2× 2.77 Å in Ba_2_Ag_4_Se_5_ and 2.59 Å - 2.65 Å in Rb_12_Nb_6_Se_35_). In Rb_12_Nb_6_Se_35_, the bond angles of approximately 164° deviate substantially from linearity, which is likely caused by the packing effect. Despite the distortion, the Se_3_^4–^ unit of Rb_12_Nb_6_Se_35_ can easily be distinguished from the Se_3_^2–^ unit occurring in the same structure, which shows bond distances of 2.39 Å and a bond angle of 94°.

While no isolated, linear hypervalent Te_3_^4–^ anion has been reported so far, the isoelectronic linear units P_3_^7–^, As_3_^7–^, Sb_3_^7–^, and Bi_3_^7–^ all occur in the numerous representatives of the Ca_14_AlSb_11_ type [46], including Ca_14-x_Eu_x_MnSb_11_, which exhibits colossal magnetoresistance [47], and the high temperature thermoelectric Yb_14_MnSb_11_ [48].

The only *Q*_4_^4–^ representative known to date is Se_4_^4–^ in K_3_CuNb_2_Se_12_ [49], which is basically an almost linear Se_3_^4–^ unit (bond angle: 166°) with an additional Se atom attached via a single bond to one end. Therefore, its ideal valence-electron number is 28, namely 22 for the Se_3_^4–^ fragment plus six for the additional Se atom. Within this model, we interpret the two bonds of the linear part as *3c-4e* bonds and the exo bond as *2c-2e*. This is in accord with the 2.73 Å and 2.54 Å distances for the (*pseudo*) collinear bonds, and the short 2.39 Å bond to the fourth Se atom with a bond angle of 93°. K_3_CuNb_2_Se_12_ is another example for a complex selenide with three topologically different Se units, namely tetrameric Se_4_^4–^, Se_2_^2–^ pairs, and isolated Se^2–^, according to (K^+^)_3_Cu^+^(Nb^5+^)_2_Se_4_^4–^(Se_2_^2–^)_3_(Se^2–^)_2_.

Representatives for *Q_n_*^4–^ with *n* = 5 exist both among selenides and tellurides. In all cases, the *Q*_5_^4–^ units contain a central (almost) linear hypervalent unit, with the two remaining *Q* atoms attached via a single bond to both ends of the linear part, like the fourth atom in Se_4_^4–^. By analogy, we conclude an ideal valence-electron number of 34, namely 22 for the *Q*_3_^4–^ fragment plus 2× six for the two additional *Q* atoms, hence *Q*_5_^4–^: 5× 6 valence-electrons (from the neutral *Q* atoms) plus four for the negative charges equals 34.

The two terminal *Q* atoms may be in *cis* or *trans* conformation. The *cis* variant is realized in the heavily distorted Se_5_^4–^ of Nb_2_Se_9_, wherein the hypervalent bonds of 2.64 Å and 2.66 Å exhibit a bond angle of only 143°, and the terminal single bonds of 2.36 Å/2.37 Å are both connected with a 83° bond angle. Considering the presence of a 2.90 Å Nb–Nb bond, Nb is most likely in the 4+ state, and noting the presence of two Se_2_^2–^ pairs per formula unit, (Nb^4+^)_2_Se_5_^4–^(Se_2_^2–^)_2_ is also a closed-shell material [50].

The *trans* conformation is only known within the tellurides, occurring in NaTe ((Na^+^)_6_Te_5_^4–^Te^2–^) [51] and Ba_2_SnTe_5_ ((Ba^2+^)_6_(Sn^4+^)_3_Te_5_^4–^(Te^2–^)_10_) [52]. An isoelectronic cationic variant is I_5_^+^ in I_5_AsF_6_ [53], while analogous pnictides have not been reported yet.

The largest representative of the series *Q_n_*^4–^ currently known is Te_7_^4–^, coordinated to an Ag^+^ or Hg^2+^ cation via the central Te and the two terminal Te atoms [54,55]. The Te_7_^4–^ anion can formally be constructed from adding two neutral Te atoms to a *cis*-Te_5_^4–^. In case of the Ag compound, the central Te_3_ unit contains hypervalent bonds of 2.87 Å and 3.23 Å with a bond angle of 174°, and the additional Te atoms form regular *2c-2e* bonds of 2.71 Å to 2.76 Å.

Two interpenetrating Te_3_^4–^ units forming an almost square planar Te_5_^6–^ unit with a central four-bonded Te atom are found in K_2_SnTe_5_ with distances of 3.02 Å and 3.06 Å [56]. These units can polymerize [57], the products of which will be discussed later in this manuscript. Adding two more Te atoms via single bonds, for example, results in bicyclic Te_7_^2–^ [58].

### 2.2. Infinite motifs in polyselenides and polytellurides

#### 2.2.1. One-dimensional motifs: chains

The above-mentioned fragments *Q_n_*^2–^ and *Q_n_*^4–^ can form higher dimensional arrays such as infinite chains, ribbons or layers. At least two bonds are required per *Q* atom in infinite chains, but the *Q* atoms may only participate in two *2c-2e* bonds, when their oxidation state is 0. Such neutral chains exist in the elements selenium and tellurium. On the other hand, Te is especially well known for its ability to form electron deficient multicenter bonds, thereby producing linear [∞1 Te^–^] chains. These chains occur in CuTe [59], UTe_2_ [60] and Ca_0.66_K_4_Te_3_ [61] with typical intrachain distances between 3.0 Å and 3.1 Å. These interactions are delocalized *2c-1e* (“half”) bonds, wherein one *p* orbital is half-filled, indicating a one-dimensional metal.

**Figure 5:**
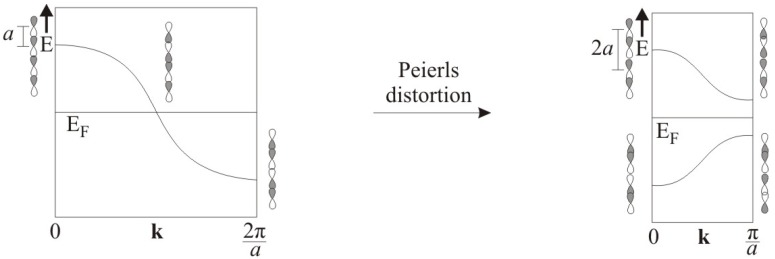
Peierls distortion of a linear [∞1 Te^–^] chain.

Such linear equidistant chains may undergo a Peierls distortion, i.e. exhibit alternating short and long distances, occurring with a metal-insulator transition (Figure 5) [62]. There are also examples of distorted linear chains of Te atoms with a formal charge of –1 such as in Cs_5_Te_3_ [63] or K_5_Te_3_ [64]. In these compounds, the Te atom chain features two different Te–Te distances, one of around 2.8 Å and the other larger than 3.5 Å. Thus, a description as [∞1 Te_2_^2–^] is more appropriate for these compounds, wherein van der Waals forces connect the pairs to linear chains.

^–^] chain.

**Figure 6:**
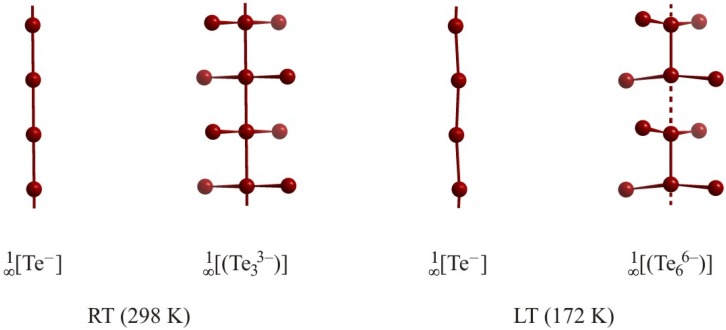
Various Te atom chains of TlTe.

The dimorph TlTe is a nice example of a material exhibiting different Te atom chains [65]. The room temperature (RT) modification of TlTe represents an equidistant [∞1 Te^–^] chain with Te–Te distances of 3.08 Å. A parallel running, second linear equidistant Te atom chain within the same structure is more complex, as two additional Te atoms are connected to each chain atom via hypervalent bonds of 3.01 Å, yielding a [∞1 Te_3_^3–^] chain (Figure 6). Thus, the RT modification may be written as (Tl^+^)_4_Te_3_^3–^Te^–^. In the low temperature (LT, 172 K) modification, both chains are distorted. Every second [∞1 Te_3_^3–^] chain is not equidistant with alternation distances of 2.86 Å and 3.30 Å, while the other [∞1 Te_3_^3–^] remain undistorted. Moreover, the linear equidistant [∞1 Te^–^] chain of the RT form is slightly bent in the LT form. Overall, the LT phase may be viewed as (Tl^+^)_16_Te_6_^6–^(Te_3_^3–^)_2_(Te^–^)_4_.

**Figure 7:**
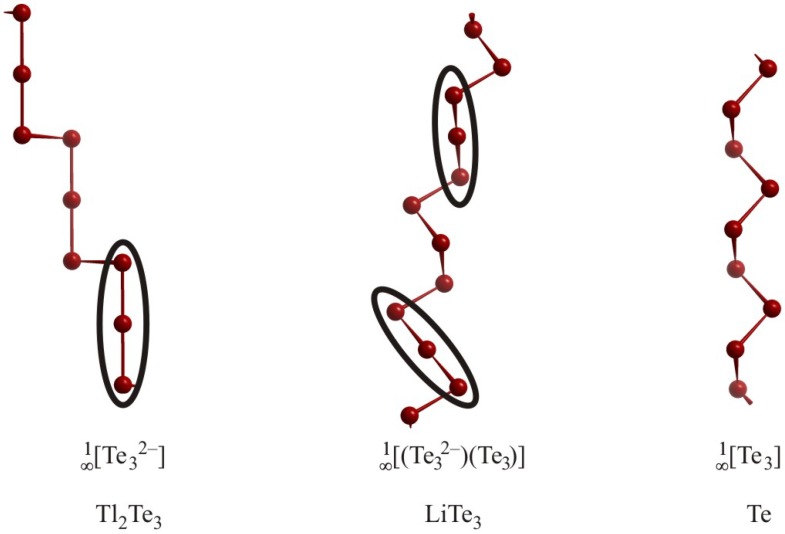
Infinite Te atom chains with different VEC(Te) in Tl_2_Te_3_ (left), LiTe_3_ (center) and Te (right).

By reducing the number of valence electrons from 7 per atom in such a one-dimensional unit, the conditions become more and more complicated. The Te atoms in Tl_2_Te_3_, for example, have a valence electron concentration (VEC(Te)) of 6⅔, forming an infinite Te chain that can be described as polymerized linear Te_3_^4–^ fragments (Figure 7). The bonds between the Te_3_^4–^ units decrease the negative charges, leading to the description [∞1 Te_3_^2–^] [66]. Two different distances were found in this chain due to different bonding situations. The short distance of 2.83 Å, a typical length for a *2c-2e* Te–Te bond, is found between the Te atoms of charge –½, the second distance (3.02 Å) occurs between Te^1–^ and Te^½–^, indicative of a *3c-4e* bond.

In LiTe_3_ [67], the VEC(Te) within the chain is lowered to 6⅓. Consequently, this chain exhibits parts known from the Te atom chains in Tl_2_Te_3_ as well as from elemental Te [68], and can therefore be viewed as [∞1 (Te_3_)(Te_3_^2–^)]. The distances within this chain are 2.85 Å for the *2c-2e* bonds in the neutral Te_3_ part of the chain, 3.02 Å for the *3c-4e* bond in the Te_3_^2–^ unit and 2.91 Å for the bond connecting these fragments.

#### 2.2.2. One-dimensional motifs: Ribbons

**Figure 8:**
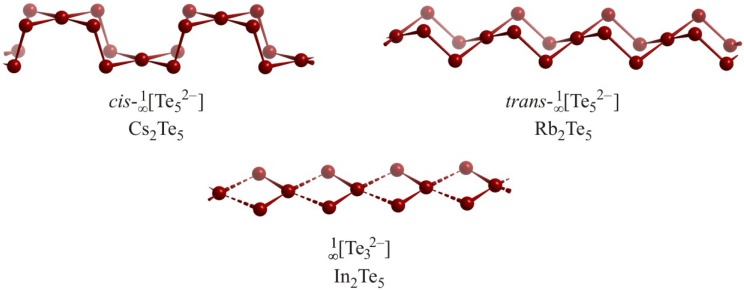
Infinite Te ribbons with different modifications in Cs_2_Te_5_ (top left), Rb_2_Te_5_ (top right) and In_2_Te_5_ (bottom center).

Only very few polychalcogenides exist that contain one-dimensionally extended *Q* atom substructures with more than two *Q*–*Q* bonds per *Q* atom. The dialkali pentatellurides show two different forms of intercondensation of above-mentioned Te_5_^6–^ squares to yield [∞1 Te_5_^2–^] ribbons with VEC(Te) = 6.4, namely the *cis* conformation in Cs_2_Te_5_ [69] and the *trans* conformation in Rb_2_Te_5_ [70] (Figure 8).

The distances in these ribbons range from 2.78 Å in Rb_2_Te_5_ and 2.77 Å in Cs_2_Te_5_ for the *2c-2e* bond between the Te_5_ units to 3.04 Å in Rb_2_Te_5_ and 3.05 Å in Cs_2_Te_5_ for the *3c-4e* bond within the Te_5_ units. In_2_Te_5_ [71] possesses a similar unit with a higher VEC(Te) of 6⅔, which causes a distortion, namely an alternation of short and long Te–Te distances of 2.83 Å (solid lines) and 3.36 Å (dashed lines). Therefore it is best described as a one-dimensional arrangement of Te_3_^2–^ fragments.

#### 2.2.3. Two-dimensional motifs: Layers

**Figure 9:**
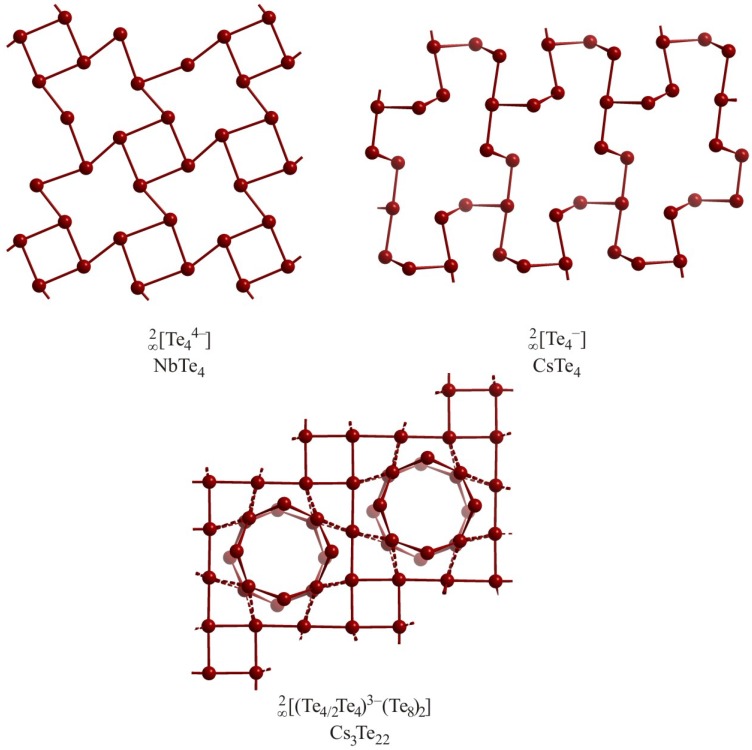
Different T nets in NbTe_4_ (top left), CsTe_4_ (top right) and Cs_3_Te_22_ (bottom center).

Compounds with hypervalently bonded Te atoms that are arranged in planar or puckered layers are often dominated by T-shaped fragments. These building blocks are then either directly connected to each other or bridged via other Te atoms. An overview of (schematic) T nets was published in the year 2004 [72]. One of the simplest examples of a T network containing Te atoms can be found in the planar layers of NbTe_4_ [73]. Each Te atom in this layer is surrounded by three other Te atoms in form of a heavily distorted T. Four-membered rings, exhibiting Te–Te distances of 3.30 Å, are connected to surrounding four-membered rings via shorter bonds of 2.88 Å (Figure 9). This hole-style arrangement in NbTe_4_ is subject to a distortion, driven by a charge density wave along the Nb atom chains perpendicular to the layer of interest. Considering the large difference of 0.4 Å between these distances, one could view this layer as loosely connected Te_2_^2–^ units. This is in contrast to the Sb atom layer of Hf_5_Sb_9_ [74], wherein all bonds are between 2.99 Å and 3.03 Å, i.e. all bonds of that T net are electron deficient multicenter bonds [75].

CsTe_4_ [76] also features T-shaped Te atom units forming a layer, which is comprised of a polymerized Te_4_^4–^ anion. This anion loses, due to this polymerization, three charges and builds a puckered layer that could be described as [∞2 Te_4_^–^]. The distances in the original T-shaped fragment in this layer are 2.92 Å and 3.14 Å for the collinear bonds, and 2.84 Å for the perpendicular bond. The connection between two (similar or different) fragments is slightly shorter (2.76 Å). The majority of the Te atoms are twofold coordinated and provide bonding angles between 96° and 103°. Therefore, the bonds within the Te_4_^4–^ fragments could be considered as asymmetric *3c-4e* bonds and the remaining bonds as *2c-2e* bonds.

Cs_3_Te_22_ provides an example of an electron-poor layer of Te atoms [77]. Cs_3_Te_22_ contains also neutral eight-membered Te rings, and can be described as (Cs^+^)_3_(Te_8_)_2_(Te_4_Te_4/2_)^3–^. Its planar [∞2 Te_4_Te_4/2_^3–^] layer consists of two- and threefold connected Te atoms, with the former being linearly coordinated and the latter T-shaped. The linearly bonded atoms interconnect the Te_4_ squares comprising the T connected Te atoms. All Te–Te distances are between 3.00 Å and 3.07 Å. Band structure calculations indicate that this layer would be semiconducting with a charge of –4, but its actual charge of –3 renders it metallic [78].

#### 2.2.4. Two-dimensional motifs: Chains connected to layers

**Figure 10:**
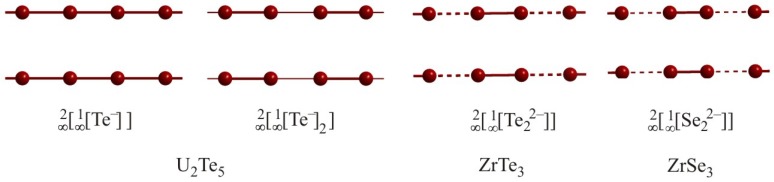
Planar layers of linear chains in U_2_Te_5_ (left), ZrTe_3_ (center) and ZrSe_3_ (right).

The previously discussed lower dimensional fragments can be connected to two-dimensional layers in several ways. The binary chalcogenides UTe_2_ [79], U_2_Te_5_ [80], α-UTe_3_ [81] and Zr*Q*_3_ [82,83] all contain linear chains aligned to form planar layers (Figure 10).

The structure of U_2_Te_5_ contains two different kinds of planar Te atom layers, i.e. [∞2[∞1 Te^–^]] and [∞2[∞1 Te^–^)_2_]]. The [∞2[∞1 Te^–^ ]] layer is sandwiched between two puckered [UTe] double layers, while two [∞2[∞1 Te^–^)_2_]] layers are separated by a van der Waals gap and sandwiched between puckered double slabs. Both layers are comprised of linear chains with interchain distances of 4.18 Å. Within the [∞2[∞1 Te^–^]] layer, almost equidistant intrachain bond lengths of 3.03 Å and 3.05 Å occur, while the [∞2[∞1 Te^–^)_2_]] layer exhibits Te_2_^2–^ pairs (2.90 Å) interconnected by longer interactions of 3.18 Å to a linear chain.

A very similar almost equidistant chain is found in UTe_2_ as well (3.05 Å and 3.08 Å), while the distortion in α-UTe_3_ is more severe with alternating distances of 2.75 Å and 3.35 Å. Similarly, the isostructural ZrTe_3_ contains Te chains with alternating intrachain distances of 2.79 Å and 3.11 Å and interchain distances of 3.93 Å. The most severe distortion is present in the analogous ZrSe_3_ with intrachain distances of 2.34 Å and 3.06 Å and an interchain distance of 3.77 Å.

Furthermore, many more complex variants, mostly tellurides, are known in this class, including UTe_5_ [84], incommensurately modulated *ALn*_3_Te_8_ (*A* = K, Rb, Cs; *Ln* = La - Nd) [85], and *Ln*SeTe_2_ (*Ln* = La - Nd, Sm) [86], which exhibit corrugated chains interconnected to planar or slightly puckered layers. A detailed discussion of all these materials would go beyond the scope of this review; square nets and their distortions were featured in a review published in 2002 [21].

#### 2.2.5. Two-dimensional motifs: oligomeric units connected to layers

**Figure 11:**
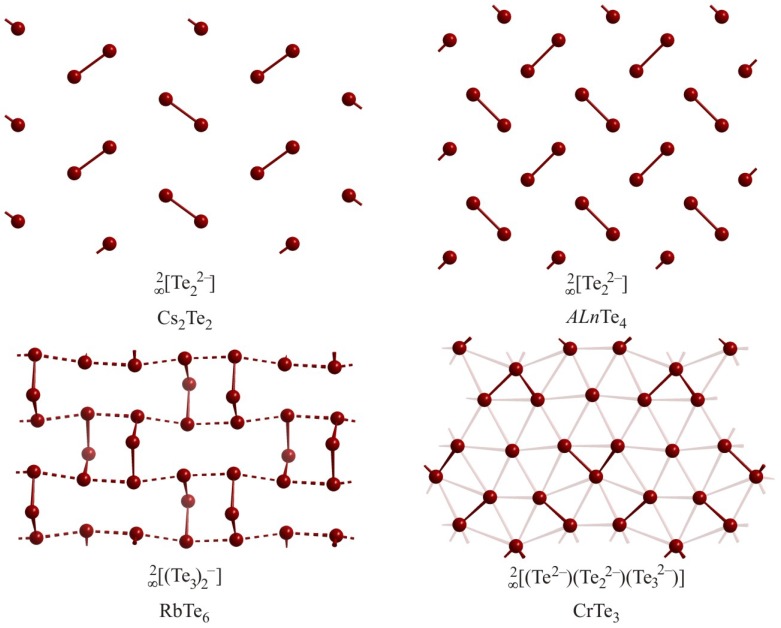
Layers of oligomeric Te atom fragments in Cs_2_Te_2_ (top left), *ALn*Te_4_ (top right), RbTe_6_ (bottom left), and CrTe_3_ (bottom right).

An enormous variety of compounds contain fragments arranged to form layers, e.g. the *Q*_2_^2–^ and *Q*_2_^3–^ units. The structure of Cs_2_Te_2_ [87] features Te_2_^2–^ dumbbells with a Te–Te distance of 2.78 Å, arranged in a herringbone-like pattern (Figure 11), and stacked between layers of cesium atoms. The distances between these dumbbells of 4.71 Å and 5.01 Å are too long for bonding interactions. Another planar layer formation only consisting of Te_2_^2–^ dumbbells (*d*_Te–Te_ = 2.78 Å) occurs in the crystal structure of *ALn*Te_4_ [88,89] with an interpair distance of 3.50 Å, which is shorter than a van der Waals contact.

The structure of RbTe_6_ [90] features a [∞2 Te_6_^–^ ] layer that is puckered because of its bent Te_3_ units, where the central Te atom takes a position either beneath or above the layer plane. Within these bent Te_3_ units, the Te–Te distances are 2.78 Å and 2.79 Å, respectively, with a bond angle of 102°, while the distances between these units are 3.20 Å and 3.21 Å. The charge of the [∞2 Te_6_^–^ ] layer cannot be readily understood; ignoring the 3.2 Å distances would indicate a charge of –2 for each Te_3_ unit, while treating them as hypervalent half bonds would ideally result in neutral Te_3_ units.

The structure of CrTe_3_ contains Te atoms in three different oxidation states in a nearly planar layer [91]. The [∞2 Te_3_^3–^] layer of this compound contains Te^2–^ ions and Te_2_^2–^ and Te_3_^2–^ units, with typical Te–Te distances of 2.82 Å. The assignment of charges is straightforward and yields a balanced formula for Cr in its +3 state: (Cr^3+^)_2_Te_3_^2–^Te_2_^2–^Te^2–^.

#### 2.2.6. Three-dimensional motifs

**Figure 12:**
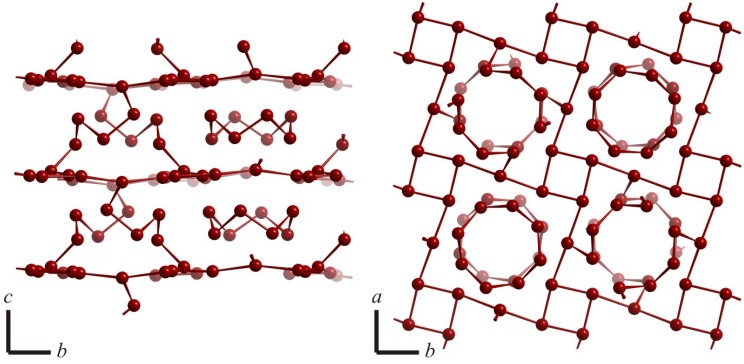
Three-dimensional network of Te atoms in Cs_4_Te_28_.

The only known compound that is comprised of a three-dimensional, covalently bonded network of Te atoms is Cs_4_Te_28_ [40]. The Te atom network is similar to the one in Cs_3_Te_22_, but in this case, half of the Te_8_ rings are broken into Te_4_ units that connect to one of the former linearly bonded atoms (Figure 12), which then assumes an oxidation state of zero.

This covalently bonded three-dimensional network could be considered as (Cs^+^)_4_Te_8_[(Te_4_)_4/2_(Te_4_Te_4/2_)_2_^4–^], wherein the neutral Te_8_ ring is comprised of typical *2c-2e* bonds (2.79 Å - 2.83 Å) like elemental sulfur. The Te–Te bonds of the connecting (also neutral) Te_4_ fragments are comparable (2.77 Å and 2.79 Å), as are the connections of these Te_4_ units of 2.80 Å with the layer of Te atoms. As a result of the connection with the Te_4_ units, all Te atoms of the layer are connected in a T-like shape exhibiting hypervalent bonds >2.9 Å, in contrast to the twofold linearly connected Te atoms in the structure Cs_3_Te_22_.

## 3. Conclusions

An overview of the variety of Se–Se and Te–Te interactions occurring in the solid state of both inorganic and organic polychalcogenides was presented. In contrast to polysulfides, the Se and Te atoms are capable of forming electron deficient multicenter (hypervalent) bonds. This adds significantly to the connectivity possibilities, e.g. the formation of T-shaped Te motifs or linear Se/Te fragments, which in turn increases the complexity of these polychalcogenides, a desired feature for, e.g., thermoelectric materials.

The tendency of Te towards higher coordination numbers is reflected in the higher abundance of complex Te atom layers, compared to Se. However, within the oligomeric units, the selenides and tellurides are quite comparable, so that more polyselenides with related two-dimensional motifs are likely to be uncovered in the near future as well.

## Figures and Tables

**Figure 4:**
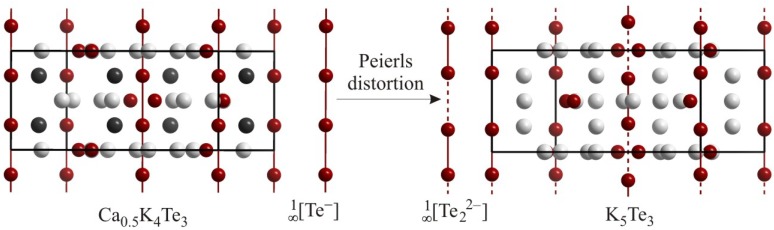
Infinite Te^1–^ chains in Ca_0.5_K_4_Te_3_ (left) and K_5_Te_3_ (right).

## References

[1] Kosbar L.L., Murray C.E., Copel M., Afzali A., Mitzi D.B. (2004). High-mobility ultrathin semiconducting films prepared by spin coating. Nature.

[2] Lange S., Nilges T. (2006). Ag_10_Te_4_Br_3_: A new silver(I) (poly)chalcogenide halide solid electrolyte. Chem. Mater..

[3] Zheng N., Bu X., Feng P. (2003). Synthetic design of crystalline inorganic chalcogenides exhibiting fast-ion conductivity. Nature.

[4] Tarascon J.-M., Armand M. (2001). Issues and challenges facing rechargeable lithium batteries. Nature.

[5] Atwood G. (2008). Phase-change materials for electronic memories. Science.

[6] Lencer D., Salinga M., Grabowski B., Hickel T., Neugebauer J., Wuttig M. (2008). A map for phase-change materials. Nat. Mater..

[7] Yamada N., Wuttig M. (2007). Phase-change materials for rewriteable data storage. Nat. Mater..

[8] Zakery A., Elliott S.R. (2003). Optical properties and applications of chalcogenide glasses: A review. J. Non-Cryst. Sol..

[9] Lowhorn N.D., Tritt T.M., Abbott E.E., Kolis J.W. (2006). Enhancement of the power factor of the transition metal pentatelluride HfTe_5_ by rare-earth doping. Appl. Phys. Lett..

[10] Rowe D.M. (2006). Thermoelectrics Handbook: Macro to Nano.

[11] Sootsman J.R., Kong H., Uher C., D’Angelo J.J., Wu C.-I., Hogan T.P., Caillat T., Kanatzidis M.G. (2008). Large enhancements in the thermoelectric power factor of bulk PbTe at high temperature by synergistic nanostructuring. Angew. Chem. Int. Ed..

[12] Xu H., Kleinke K.M., Holgate T., Zhang H., Su Z., Tritt T.M., Kleinke H. (2009). Thermoelectric performance of Ni_y_Mo_3_Sb_7-x_Te_x_ (y ≤ 0.1, 1.5 ≤ x ≤ 1.7). J. Appl. Phys..

[13] Böttcher P., Getzschmann J., Keller R. (1993). Zur Kenntnis der Dialkalimetalldichalkogenide β-Na_2_S_2_, K_2_S_2_, α-Rb_2_S_2_, β-Rb_2_S_2_, K_2_Se_2_, Rb_2_Se_2_, α-K_2_Te_2_, β-K_2_Te_2_ und Rb_2_Te_2_. Z. Anorg. Allg. Chem..

[14] Schäfer H., Eisenmann B., Müller W. (1973). Zintl Phases: Transitions between metallic and ionic bonding. Angew. Chem. Int. Ed. Engl..

[15] Nesper R. (1990). Zintl-phases containing Li. Prog. Solid State Chem..

[16] Kauzlarich S.M. (1996). Chemistry, Structure, and Bonding of Zintl Phases and Ions.

[17] Siegel S.G. (1963). Crystallographic studies of XeF_2_ and XeF_4_. J. Am. Chem. Soc..

[18] Curnow O.J. (1998). A Simple qualitative molecular-orbital/valence-bond description for the bonding in main group "hypervalent" molecules. J. Chem. Educ..

[19] Papoian G. A., Hoffmann R. (2000). Hypervalent bonding in one, two, and three dimensions: Extending the Zintl-Klemm concept to nonclassical electron-rich networks. Angew. Chem. Int. Ed..

[20] Böttcher P. (1988). Tellurium-Rich Tellurides. Angew. Chem. Int. Ed. Engl..

[21] Patschke R., Kanatzidis M.G. (2002). Polytelluride compounds containing distorted nets of tellurium. Phys. Chem. Chem. Phys..

[22] Xu J., Kleinke H. (2008). Unusual Sb–Sb bonding in high temperature thermoelectric materials. J. Comput. Chem..

[23] Kanatzidis M.G. (1995). From cyclo-Te_8_ to Te_x_^n-^ Sheets: Are Nonclassical Polytellurides More Classical than We Thought?. Angew. Chem. Int. Ed. Engl..

[24] Böttcher P., Doert T. (1998). Chalcogen-rich chalcogenides: from the first ideas to a still growing field of research. Phosphorus, Sulfur, Silicon.

[25] Smith D.M., Ibers J.A. (2000). Syntheses and solid-state structural chemistry of polytelluride anions. Coord. Chem. Rev..

[26] Föppl H., Busmann E., Frorath F.K. (1962). Die Kristallstrukturen von α-Na_2_S_2_ und K_2_S_2_, β-Na_2_S_2_ und Na_2_Se_2_. Z. Anorg. Allg. Chem..

[27] Batchelor R.J., Einstein F.W.B., Gay I.D., Jones C.H.W., Sharma R.D. (1993). Syntheses and solid-state NMR of tetrabutylammonium hydrogen telluride, tetramethylammonium hydrogen selenide and bis(tetramethylammonium) ditelluride and x-ray crystal structures of Me_4_NSeH and (Me_4_N)_2_Te_2_. Inorg. Chem..

[28] Thiele K.-H., Steinicke A., Dümichen U., Neumüller B. (1996). Darstellung und Reaktionen von Natriumtellurid, Na_2_Te - Kristallstruktur von [Na(CH_3_OH)_3_]_2_Te_2_. Z. Anorg. Allg. Chem..

[29] Pauling L. (1948). The Nature of the Chemical Bond.

[30] Böttcher P. (1977). Die Kristallstruktur von K_2_S_3_ und K_2_Se_3_. Z. Anorg. Allg. Chem..

[31] Assoud A., Soheilnia N., Kleinke H. (2004). Band gap tuning in new strontium seleno-stannates. Chem. Mater..

[32] Assoud A., Soheilnia N., Kleinke H. (2005). The new semiconducting polychalcogenide Ba_2_SnSe_5_ exhibiting Se_3_^2-^ units and distorted SnSe_6_ octahedra. J. Solid State Chem..

[33] Eisenmann B., Schäfer H. (1978). K_2_Te_3_: The first binary alkali-metal polytelluride with Te_3_^2-^ ions. Angew. Chem. Int. Ed. Engl..

[34] Cui Y., Assoud A., Xu J., Kleinke H. (2007). Structures and Physical Properties of new Semiconducting gold and copper polytellurides: Ba_7_Au_2_Te_14_ and Ba_6.76_Cu_2.42_Te_14_. Inorg. Chem..

[35] Getzschmann J.R., Rönsch E., Böttcher P. (1997). Crystal structure of dinatriumtetraselenide, Na_2_Se_4_. Z. Kristallogr. -NCS.

[36] Huffman J.C., Haushalter R.C. (1984). Preparation and crystal structure of (Ph_4_P)_2_Te_4_·2CH_3_OH. Z. Anorg. Allg. Chem..

[37] Müller V., Frenzen G., Dehnicke K., Fenske D. (1992). Synthese, FIR-Spektren und Kristallstrukturen der Pentaselenide K_2_Se_5_ und (Na(15-Krone-5))_2_Se_5_. Z. Naturforsch. B.

[38] Weller F., Adel J., Dehnicke K. (1987). Polyselenide mit langkettigen Tetraalkylammoniumionen. Die Kristallstruktur von Trimethyl-tetradecyl-ammonium-hexaselenid. Z. Anorg. Allg. Chem..

[39] Warren C.J., Haushalter R.C., Bocarsly A.B. (1996). Electrochemical synthesis of a pseudo-two-dimensional polytelluride containing Te_12_^2-^ anions: Structure of [(C_2_H_5_)_4_N]_2_Te_12_. J. Alloys Compd..

[40] Sheldrick W.S., Wachhold M. (1996). Synthesis and structure of Cs_2_Te_13_ and Cs_4_Te_28_, tellurium-rich tellurides on the methanolothermal route to Cs_3_Te_22_. Chem. Commun..

[41] Assoud A., Xu J., Kleinke H. (2007). Structures and physical properties of new semiconducting polyselenides Ba_2_Cu_δ_Ag_4-__δ_Se_5_ with unprecedented linear Se_3_^4-^ units. Inorg. Chem..

[42] Dürichen P., Bolte M., Bensch W. (1998). Synthesis, crystal structure, and properties of polymeric Rb_12_Nb_6_Se_35_, a novel ternary niobium selenide consisting of infinite anionic chains built up by Nb_2_Se_11_ units containing an uncommon Se_3_^4-^-fragment. J. Solid State Chem..

[43] Tasman H.A., Boswijk K.H. (1955). Reinvestigation of the crystal structure of CsI_3_. Acta Crystallogr..

[44] Mooney-Slater R.C.L. (1959). The triiodide ion in tetraphenylarsonium triiodide. Acta Crystallogr..

[45] Rundle R.E. (1963). On the Problem Structure of XeF_4_ and XeF_2_. J. Am. Chem. Soc..

[46] Cordier G., Schäfer H., Stelter M. (1984). Darstellung und Struktur der Verbindung Ca_14_AlSb_11_. Z. Anorg. Allg. Chem..

[47] Kim H., Olmstead M.M., Klavins P., Webb D.J., Kauzlarich S.M. (2002). Structure, magnetism, and colossal magnetoresistance (CMR) of the ternary transition metal solid solution Ca_14-x_Eu_x_MnSb_11_ (0 < x <14). Chem. Mater..

[48] Brown S.R., Kauzlarich S.M., Gascoin F., Snyder G.J. (2006). Yb_14_MnSb_11_: New high efficiency thermoelectric material for power generation. Chem. Mater..

[49] Lu Y., Ibers J.A. (1991). Synthesis and characterization of the new quaternary one-dimensional chain materials, potassium copper niobium selenides, K_2_CuNbSe_4_ and K_3_CuNb_2_Se_12_. Inorg. Chem..

[50] Sunshine S.A., Ibers J.A. (1987). Redetermination of the structures of CuTaS_3_ and Nb_2_Se_9_. Acta Crystallogr. C.

[51] Böttcher P., Keller R. (1985). The crystal structure of NaTe and its relationship to tellurium-rich tellurides. J. Less-Common Met..

[52] Assoud A., Derakhshan S., Soheilnia N., Kleinke H. (2004). Electronic structure and physical properties of the semiconducting polytelluride Ba_2_SnTe_5_ with a unique Te_5_^4-^ unit. Chem. Mater..

[53] Apblett A., Grein F., Johnson J.P., Passmore J., White P.S. (1986). Preparation and X-ray crystal structure of [I_5_^+^][AsF_6_^-^], an electronic structure of the I_5_^+^ cation. Inorg. Chem..

[54] McConnachie J.M., Ansari M.A., Bollinger J.C., Salm R.J., Ibers J.A. (1993). Synthesis and structural characterization of the telluroargentate [PPh_4_]_2_[NEt_4_][AgTe_7_] and telluromercurate [PPh_4_]_2_[HgTe_7_] compounds containing the unprecedented η_3_-Te_7_^4-^ polytelluride anion. Inorg. Chem..

[55] Smith D.M., Roof L.C., Ansari M.A., McConnachie J.M., Bollinger J.C., Pell M.A., Salm R.J., Ibers J.A. (1996). Synthesis, reactivity, and structural characterization of the nonclassical [MTe_7_]^n-^ Anions (M = Ag, Au, n = 3; M = Hg, n = 2). Inorg. Chem..

[56] Eisenmann B., Schwerer H., Schäfer H. (1983). Plane, zu Ketten verknüpfte Te_5_^6-^-Anionen im K_2_SnTe_5_. Mat. Res. Bull..

[57] Bernstein J., Hoffmann R. (1985). Hypervalent Tellurium in One-Dimensional Extended Structures Containing Te_5_^n-^ Units. Inorg. Chem..

[58] Harbrecht B., Selmer A. (1994). Rhenium selenide tellurides Re_2_Se_x_Te_5-x_: The structure of Re_6_Se_8_Te_7_. Z. Anorg. Allg. Chem..

[59] Anderko K., Schubert K. (1954). Untersuchungen im System Kupfer-Tellur. Z. Metallk..

[60] Klein-Haneveld A.J., Jellinek F. (1970). The crystal structure of stoichiometric uranium ditelluride. J. Less-Common Met..

[61] Schewe-Miller I., Böttcher P. (1992). Ternäre Telluride mit W_5_Si_3_-Typ-Struktur: M_x_K_4_Te_3_ (M=Ca, Sr). J. Alloys Compd..

[62] Peierls R.E. (1955). Quantum Theory of Solids.

[63] Schewe-Miller I., Böttcher P. (1991). Synthesis and crystal structures of K_5_Se_3_, Cs_5_Te_3_ and Cs_2_Te. Z. Kristallogr..

[64] Schewe-Miller I., Böttcher P. (1990). Darstellung und Kristallstruktur des K_5_Te_3_. Z. Naturforsch. B.

[65] Stöwe K. (2000). The Phase Transition of TlTe: Crystal Structure. J. Solid State Chem..

[66] Doert T., Cardoso Gil R.H., Böttcher P. (1999). The crystal structure of Tl_2_Te_3_ - a reinvestigation. Z. Anorg. Allg. Chem..

[67] Valentine D.Y., Cavin O.B., Yakel H.L. (1977). On the crystal structure of LiTe_3_. Acta Crystallogr. B.

[68] Bradley A.J. (1924). The crystal structure of Te. Philos. Mag..

[69] Böttcher P., Kretschmann U. (1982). Darstellung und Kristallstruktur von Dicaesiumpentatellurid Cs_2_Te_5_. Z. Anorg. Allg. Chem..

[70] Böttcher P., Kretschmann U. (1983). Darstellung und Kristallstruktur von Dirubidiumpentatellurid, Rb_2_Te_5_. J. Less-Common Met..

[71] Sutherland H.H., Hogg J.H.C., Walton P.D. (1976). Indium polytelluride In_2_Te_5_. Acta Crystallogr. B.

[72] Ienco A., Proserpio D. M., Hoffmann R. (2004). Main group element nets to a T. Inorg. Chem..

[73] Böhm H., von Schnering H.G. (1985). The modulated structure of niobium tetratelluride NbTe_4_. Z. Kristallogr..

[74] Assoud A., Kleinke K.M., Soheilnia N., Kleinke H. (2004). T-shaped nets of Sb atoms in the binary antimonide Hf_5_Sb_9_. Angew. Chem. Int. Ed..

[75] Xu J., Kleinke K.M., Kleinke H. (2008). electronic structure and physical properties of Hf_5_Sb_9_ containing a unique T net of Sb atoms. Z. Anorg. Allg. Chem..

[76] Böttcher P., Kretschmann U. (1985). Darstellung und Kristallstruktur von CsTe_4_. Z. Anorg. Allg. Chem..

[77] Sheldrick W.S., Wachhold M. (1995). Discrete crown-shaped Te_8_ rings in Cs_3_Te_22_. Angew. Chem. Int. Ed. Engl..

[78] Liu Q., Goldberg N., Hoffmann R. (1996). A 2,3-connected tellurium net and the Cs_3_Te_22_ phase. Chem. Eur. J..

[79] Stöwe K. (1996). Contributions to the crystal chemistry of uranium tellurides. III. Temperature-dependent structural investigations on uranium ditelluride. J. Solid State Chem..

[80] Stöwe K. (1996). Beiträge zur Kristallchemie der Urantelluride. II. Die Kristallstruktur des Diuranpentatellurids U_2_Te_5_. Z. Anorg. Allg. Chem..

[81] Stöwe K. (1996). Beiträge zur Kristallchemie der Urantelluride. I. Die Kristallstruktur des α-Urantritellurids. Z. Anorg. Allg. Chem..

[82] Krönert W., Plieth K. (1965). Die Struktur des Zirkontriselenids ZrSe_3_. Z. Anorg. Allg. Chem..

[83] Felser C., Finckh E.W., Kleinke H., Rocker F., Tremel W. (1998). Electronic properties of ZrTe_3_. J. Mater. Chem..

[84] Noel H. (1985). Crystal structure of the low-dimensional uranium pentatulluride: UTe_5_. Inorg. Chim. Acta.

[85] Patschke R., Heising J., Schindler J. L., Kannewurf C. R., Kanatzidis M. (1998). Site occupancy wave and unprecedented infinite zigzag (Te_2_^2-^)_n_ chains in the flat Te nets of the new ternary rare earth telluride family. J. Solid State Chem..

[86] Fokwa B.P.T., Doert T. (2005). The ternary rare-earth polychalcogenides LaSeTe_2_, CeSeTe_2_, PrSeTe_2_, NdSeTe_2_, and SmSeTe_2_: Syntheses, crystal structures, electronic properties, and charge-density-wave-transitions. Solid State Sci..

[87] Getzschmann J., Böttcher P., Kaluza W. (1996). Darstellung und Kristallstrukturen von β-Rb_2_Te_2_ und Cs_2_Te_2_ sowie die Verfeinerung der Strukturen von Ca_2_P_2_ und Sr_2_As_2_. Z. Kristallogr..

[88] Dürichen P., Bensch W. (1997). Cesium gadolinium tetratelluride. Acta Crystallogr. C.

[89] Stöwe K. (2003). Syntheses and crystal structures of KPrTe_4_, KGdTe_4_ and RbGdTe_4_. Solid State Sci..

[90] Sheldrick W.S., Schaaf B. (1994). RbTe_6_, ein Polytellurid mit Schichtstruktur [Te_6_^-^]. Z. Naturforsch. B.

[91] Klepp K.O., Ipser H. (1982). CrTe_3_ - A novel transition-Metal polytelluride. Angew. Chem. Int. Ed. Engl..

